# Strengthening individual capacity in monitoring and evaluation of malaria control programmes to streamline M&E systems and enhance information use in malaria endemic countries

**DOI:** 10.1186/s12936-016-1354-y

**Published:** 2016-05-28

**Authors:** Ashley Garley, Erin Eckert, Ali Sie, Maurice Ye, Keziah Malm, Edwin A. Afari, Mamadou Sawadogo, Samantha Herrera, Elizabeth Ivanovich, Yazoume Ye

**Affiliations:** MEASURE Evaluation, ICF International, 530 Gaither Road, Suite 500, Rockville, MD 20850 USA; President’s Malaria Initiative, US Agency for International Development, Washington DC, USA; Centre de Recherche en Santé de Nouna, CRSN, Ouagadougou, Burkina Faso; School of Public Health, University of Legon, Accra, Ghana; National Malaria Control Programme, Accra, Ghana; Unité de formation et de recherche, Science de la Santé, Centre de recherche international pour la Santé, Université de Ouagadougou, Ouagadougou, Burkina Faso; United Nations Foundation, Washington DC, USA

**Keywords:** Capacity building, Monitoring and evaluation, Health information systems, Malaria, Training, Ghana, Burkina Faso

## Abstract

**Background:**

Malaria control interventions in most endemic countries have intensified in recent years and so there is a need for a robust monitoring and evaluation (M&E) system to measure progress and achievements. Providing programme and M&E officers with the appropriate skills is a way to strengthen malaria’s M&E systems and enhance information use for programmes’ implementation. This paper describes a recent effort in capacity strengthening for malaria M&E in sub-Saharan Africa (SSA).

**Methods:**

From 2010 to 2014, capacity-strengthening efforts consisted of organizing regional in-person workshops for M&E of malaria programmes for Anglophone and Francophone countries in SSA in collaboration with partners from Ghana and Burkina Faso. Open-sourced online courses were also available in English. A post-workshop assessment was conducted after 5 years to assess the effects of these regional workshops and identify gaps in capacity.

**Results:**

The regional workshops trained 181 participants from 28 countries from 2010 to 2014. Trained participants were from ministries of health, national malaria control and elimination programmes, non-governmental organizations, and development partners. The average score (%) for participants’ knowledge tests increased from pretest to posttest for Anglophone workshops (2011: 59 vs. 76, 2012: 41 vs. 63, 2013: 51 vs. 73; 2014: 50 vs. 74). Similarly, Francophone workshop posttest scores increased, but were lower than Anglophone due to higher scores at pretest. (2011: 70 vs. 76, 2012: 74 vs. 79, 2013: 61 vs. 68; 2014: 64 vs. 75). Results of the post-workshop assessment revealed that participants retained practical M&E knowledge and skills for malaria programs, but there is a need for a module on malaria surveillance adapted to the pre-elimination context.

**Conclusion:**

The workshops were successful because of the curriculum content, facilitation quality, and the engagement of partner institutions with training expertise. Results from the post-workshop assessment will guide the curriculum’s development and restructuring for the next phase of workshops. Country-specific malaria M&E capacity needs assessments may also inform this process as countries reduce malaria burden.

## Background

In recent years, heightened commitments from governments and several partners to eliminate malaria have resulted in increased funding globally for malaria [1, 2]. Governments and funding partners are both requesting robust evidence for the health returns on their investments. To generate such evidence, countries need to have a solid information system in place for monitoring malaria interventions and measuring achievements [3, 4]. However, there are important challenges in the performance of existing information systems at the country level, particularly in their ability to provide timely and quality data to inform malaria programme implementation [1, 2, 5, 6].

At the global level, donors often use survey-based data to evaluate the impact of their programmes, in terms of coverage, morbidity, and mortality [7, 8]. These data are generally robust and reliable over time. However, these data are not timely and do not provide the granular level of detail that programme managers need to monitor programme implementation. This situation can create disconnect between various stakeholders regarding the status of programme implementation. Building capacity to collect, analyse, and use data from multiple information sources is a critical need within disease control programmes. There are different means to build this M&E capacity, including degree programmes, on-the-job training, and short-term certificate courses [9–11]. This paper examines the importance of short-term training in building capacity for M&E within National Malaria Control Programmes.

Degree training builds skills using a long-term approach, whereby individuals take multiple courses to complete a degree programme. However, the degree training approach is not always feasible for adult learning due to high costs and the amount of time away from work needed to complete the degree [12, 13]. On-the-job training through regular supportive supervision is a mentoring approach that involves direct contact between a supervisor and an individual, and this training builds skills on the job [14, 15]. However, this approach can also vary greatly depending on the level of support and the relationship between the supervisor and the individual.

Short courses (in person or online) are designed to build skills quickly through intensive short-term training that usually lasts only a few weeks. This method trains many people in a short time by focusing on a narrowed topic. It is ideal for working professionals who need to build skills quickly but cannot leave their work for a longer-degree programme. Short courses often focus less on theory and include practical and applicable content and exercises that can be applied immediately to the job. Short courses are often packaged to be more affordable than lengthy degree training programmes and open-ended routine supervision [13, 16].

**Fig. 1:**
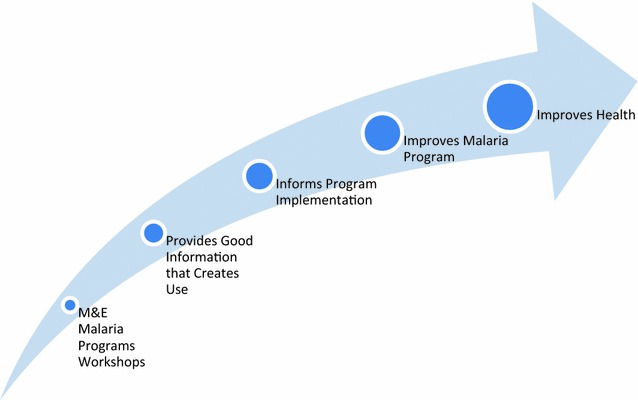
Framework for malaria M&E Anglophone and Francophone regional workshops

In 2009, a suggestion by the Roll Back Malaria (RBM) Monitoring and Evaluation Reference Group (MERG) initiated the idea of conducting a series of regional workshops for M&E of malaria programmes to strengthen countries’ capacity in developing and implementing malaria M&E plans. Using the short-course approach, under the auspices of MERG with regional partners, regional malaria M&E workshops for Anglophone and Francophone countries in SSA were designed and implemented from 2010 to 2014. The aim was to improve the quality of data and information and to help guide programme implementation and thereby reduce the malaria burden and improve health (Fig. 1). After the workshops, participants were expected to return to their malaria programmes and apply their new M&E knowledge and skills to improve their programmes’ active M&E plans and streamline the implementation of national plans.

This paper describes the process of implementing these workshops, highlights achievements, and discusses lessons learned and new challenges.

### Workshop implementation process

#### Implementing partners and target audience

Implementing partners were selected through a competitive bidding process that included evaluating organization experience, personnel, logistic capacity, and budget. The University of Ghana, School of Public Health (UGSPH) in Accra, Ghana, was chosen to implement the Anglophone workshop in 2010. The Centre de Recherche en Santé de Nouna (CRSN) and the Centre de Recherche International en Santé (CRIS) were selected to implement the Francophone workshop in Ouagadougou, Burkina Faso, in 2011. Workshop objectives aimed to build upon existing regional resources and increase regional capacity for M&E of malaria by teaching participants the M&E fundamentals specified for malaria programmes while providing hands-on experience in designing malaria M&E plans.

The workshops targeted national- and subnational-level malaria personnel, especially those responsible for gathering, analysing, and using programme-related data. Also included were personnel from non-governmental organizations (NGOs), universities, and development partners, such as USAID and Global Fund, who are responsible for the oversight of malaria programmes, especially in the areas of reporting, monitoring, and evaluation.

### Curriculum design

The curriculum was designed to meet the needs of programme managers and fill the gaps in basic monitoring and evaluation skills at the programme level. Modules were selected to provide practical tools and techniques that participants could apply to their current work. The curriculum focused on creating a good M&E plan and implementing it as a guide for malaria programmes. The curriculum was created in English and then translated to French after the 1st year for the Francophone countries. Over time, the content has been updated in line with the emerging needs for malaria control and addressing feedback from participants. These updates added new malaria interventions to stay abreast of new methods and tools used in the field. Facilitators from partner institutions contributed to the curriculum updates based on their expertise and experience. Table 1 describes the curriculum’s objectives and the benefits to participants by module.

**Table 1:** Content and objectives of the malaria M&E Anglophone and Francophone regional workshops

Module name	Objectives	Benefit to participant
Overview of malaria in SSA	Describes epidemiology of malaria in SSA, burden of malaria, global efforts for control, pre-elimination, and elimination, and various types of interventions. Module updated annually to reflect current data from annual World Malaria Reports	Provides knowledge of malaria epidemiology, current global targets and new trends and interventions
Basic concepts of M&E	Defines programme components, key concepts, and purpose of M&E	Provides correct understanding of malaria M&E malaria terminology
Role of data in decision making	Raises awareness of importance of using data to inform decisions, discussed strategies for overcoming barriers for decision-making, learned strategies for using data in programme management, implementation, and decision-making	Provides practical explanation of the importance and usefulness of M&E for a malaria programme
Designing and implementing an M&E plan	Describes functions and main elements of an M&E plan. Describes the process and implementation of a plan and discusses well-known challenges	Delivers tools and resources for designing an M&E plan
Frameworks	Identifies conceptual, results, and logical frameworks, and logic models. Defines goals and objectives for specific intervention programmes. Designs frameworks and discusses how they are used	Provides the importance and usefulness of various frameworks
Indicators	Discusses design of good quality indicators. Teaches how to critique indicators. Links indicators to frameworks and introduces indicator reference sheets	Provides the importance of indicators and how they fit in the broad view of malaria M&E
Data sources and systems	Identifies various types of data sources, including routine and non-routine sources. Discusses strengths and weaknesses of data sources, linking sources, and recognizing appropriate sources for measuring malaria intervention coverage and impact	Explains various data sources and their importance and usefulness
Data quality	Identifies data quality issues at each step of a data management system. Highlights key criteria used to assess data quality and identifies steps for ensuring data quality at all levels of the data management system. Discusses key elements of a data quality assessment	Explains the importance of data quality in the improvement of the health information system
Evaluation designs	Describes evaluation terminology, causality, internal and external validity. Teaches various types of evaluations and discusses strengths and limitations of study designs. Teaches participants how to develop an evaluation framework and select a study design that fits the purpose of a given evaluation. Includes current examples of evaluations conducted by facilitators	Offers various evaluation methods and detailed examples of current evaluations
Data management	Identifies general rules of data management. Defines roles and responsibilities and utilizes information to implement a system for good data management	Provides tools for correctly managing a health information system
Data presentation, interpretation and use	Discusses different ways to summarize data and choose the best graphic for the audience. Focuses on ensuring graphics are self-explanatory, clear, concise, and attractive, so data is easily interpreted and used	Teaches practical techniques for presenting, interpreting and using data

### Running the workshops

The workshops were 10-day courses at an academic institution. USAID/PMI provided core funding for curriculum development, start-up costs, trainers’ training, and a few competitive annual fellowships. Participants paid their course fees and travel expenses. The Anglophone course took place at the UGSPH, while the Francophone course was held at the University of Ouagadougou, School of Medicine. Teaching approaches included plenary sessions, discussions, and a group project. Facilitators led individual and group exercises, such as case studies, hands-on data analysis, data presentation exercises, and fieldwork.

Group work was a significant component of the courses. Participants were divided into groups at the beginning of each workshop and assigned to use a step-by-step approach in developing an M&E plan focused on a specific malaria control intervention. The groups developed problem statements, goals, and objectives; designed a conceptual framework; conducted strengths, weaknesses, opportunities, and threats (SWOT) analysis; chose relevant indicators for their specific malaria intervention; selected an evaluation method; and budgeted their M&E plan. Groups presented two progress reports and received feedback from peers and facilitators. On the last day of the workshop, each group presented an M&E plan.

Training materials were developed, taught, and updated annually by project staff and the local implementing universities: UGSPH, and CRSN/CRIS. In an effort to build capacity within the hosting institutions, each module was co-facilitated by one instructor from the implementing partner and another from the project staff. The number of modules taught by implementing partners gradually increased as staff became more comfortable in conducting the sessions. In the 2010 Anglophone course, five sessions were taught exclusively by project staff; however, by 2014, this was reduced to one session. In contrast, the number of sessions taught by UGSPH and in-country partners increased from 9 of 15 sessions in 2010 to 8 of 11 in 2014. Similarly, the number of modules taught exclusively by CRSN or in-country partners at the Francophone course increased from 11 of 16 sessions in 2011–12 of 16 in 2014.

### Workshop evaluation

Several evaluation tools were used to assess the workshops’ implementation and measure the knowledge gained by the participants. These tools included a module assessment form, a group work assessment form, and a workshop evaluation form [17, 18].

At the end of each module, the participants rated the quality of the content and the quality of the instruction on a scale of 1 (poor) to 10 (excellent) using the module assessment form. Facilitators reviewed the evaluations daily and made necessary improvements based on suggestions for subsequent modules.

At the end of the workshop, the participants completed the workshop assessment form, which provided feedback on content, quality of facilitation, materials, training environment, relationships between participants, and group work. Findings from this assessment provided detailed information for a variety of future workshop improvements.

In 2014, a form was introduced for peer assessment of the group work. Peers assessed group work on presentation quality, form, content, presenter explanations, and each group’s response to questions. Teamwork and general group organization were also evaluated. Scores were weighted gradually with the lowest weight assigned to the first presentation and the highest weight to the final presentation. Average scores were calculated and the winning group was recognized on the final day.

To assess knowledge gained, the participants took pre- and post-module knowledge tests on the first and last day of each workshop. These tests consisted of a series of 20 multiple-choice questions on malaria M&E content in the workshop modules.

### Overall post-training assessment

In early 2015, the training team commissioned an independent post-workshop assessment to assess the regional M&E capacity of malaria workshops from 2010 to 2014, identify gaps in capacity, and evaluate the effects of the regional training course. Specific objectives included assessing skill and knowledge retention; documenting the use of the skills and knowledge gained in the participants’ current work, and understanding how the workshop can be improved for future participants. The methodology included a document review of workshop materials, a literature review, and the design of a survey protocol with specific surveys in French and English for participants who completed the workshop, their supervisors, and stakeholders interested in malaria M&E. One hundred and eighty-one workshop participants (120 Anglophones and 61 Francophones) and 40 supervisors (21 Anglophones and 19 Francophones) were identified and contacted for an online survey, while 33 stakeholders were also contacted for an in-depth interview.

### Workshop outputs

#### Participants trained

**Fig. 2:**
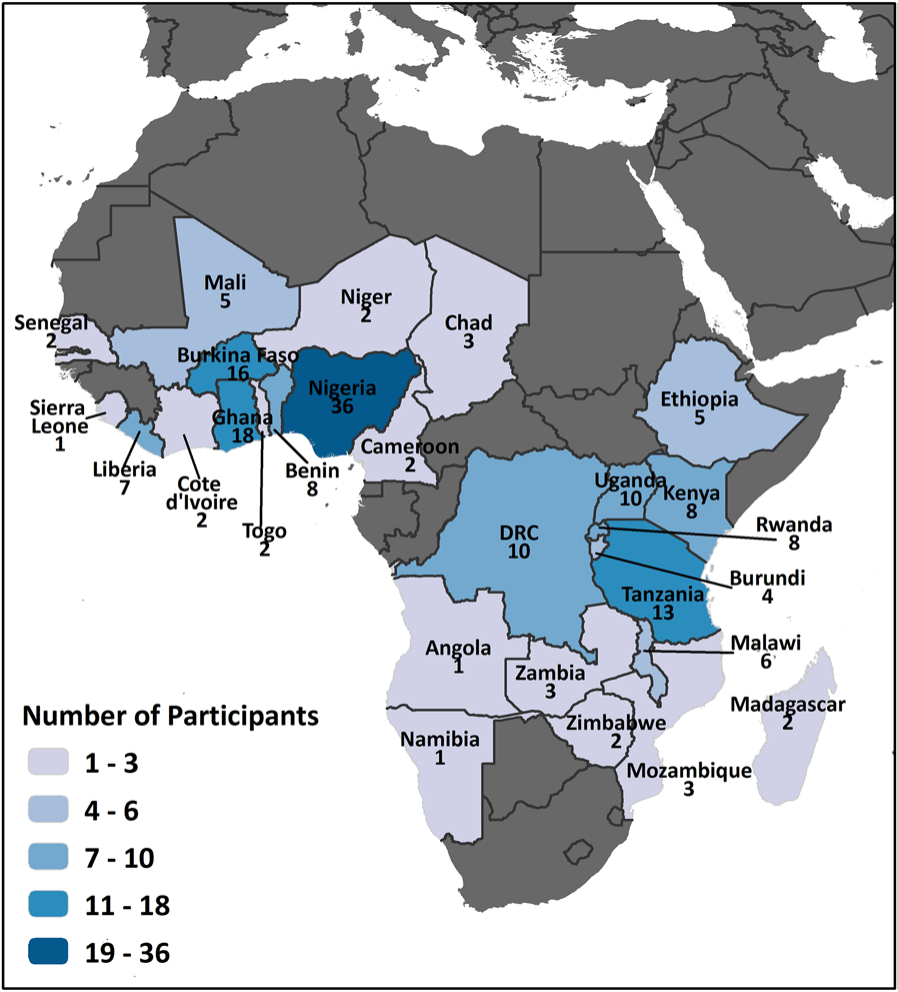
Participants trained in malaria M&E through regional workshops by country, 2011–2014

One hundred and eighty-one people were trained from 2010 to 2014. Participants came from 28 countries, with the majority from Nigeria (36), Ghana (18), and Burkina Faso (16). Tanzania (13), Democratic Republic of Congo (10) and Uganda (10) also had high participation (see Fig. 2). All participants came from SSA, except for one participant from the United States. At the time of the workshops, 41 % of the participants trained were from non-governmental organizations, 27 % worked for a ministry of health, and 22 % worked specifically for national malaria control and elimination programmes. Development partners, such as USAID and Global Fund, represented 8 % of participants trained.

#### Knowledge gained

Upon completion of the workshop, participants understood fundamental concepts and practical approaches to the M&E of malaria programmes. They learned programmatic applications of tools and data systems used to monitor and evaluate malaria programmes. The workshops provided an opportunity for hands-on experience in developing M&E plans. The results of the pre- and post-tests conducted on the first and last days of the workshop indicated the knowledge gained, and the average score was higher on the post-test compared to the pre-test for both Anglophone and Francophone workshops in all years (Table 2).

**Table 2:** Participants’ knowledge gain assessed by pre and post-test of the regional workshops, 2011–2014

Year	Anglophone		Francophone	
	Pre-test % (range)	Post-test % (range)	Pre-test % (range)	Post-test % (range)
2011	59.3 (27.5–82.5)	76.0 (35.0–95.0)	70.9 (50.0–87.5)	75.8 (60.0–92.5)
2012	41.2 (12.6–61.1)	63.0 (44.7–88.4)	74.3 (50.0–93.3)	79.4 (60.8–88.3)
2013	51.0 (23.0–77.0)	73.0 (47.0–88.0)	61.1 (42.3–73.1)	67.6 (0.0–84.6)
2014	49.8 (17.0–85.0)	74.2 (45.0–90.0)	63.7 (46.2–92.3)	75.4 (57.9–96.2)

#### Quality of the workshops

The average score per year of quality of content was above 8.50, with the highest score of 9.21 in 2014 in the Francophone course. The Anglophone course showed improvement in the quality of content from an average score of 8.11 in 2010 to 8.74 in 2014. Similarly, the rating of the quality of facilitation improved, regardless of the implementation of co-facilitation and partner-led facilitation.

#### Post-workshop effects

The use of knowledge gained during the workshop was documented in six-month and one-year follow-up qualitative surveys. The findings suggested that the M&E workshop contributed to, or helped, alumni in their work or research. Specific areas that participants noted included designing an M&E plan; defining and reporting indicators; using data for decision-making; and data collection, analysis, and feedback of data results. Workshop alumni also mentioned contributing to malaria indicator surveys (MIS) and impact evaluations.

Newly trained participants were admitted to a network of previously trained M&E professionals and invited to join the Roll Back Malaria M&E listserv [19]. These tools, moderated by MEASURE Evaluation, provide opportunities for trained participants to share ideas after the workshop, participate in M&E-specific webinars, and collaborate with others internationally in the field of M&E.

The success of these regional M&E malaria workshops has generated interest in country-specific workshops. These have taken place in Kenya (2011) and the Democratic Republic of Congo (2013 and 2015). Other countries, including Burkina Faso, Liberia, Senegal, and Tanzania, have also expressed interest. In addition, regional workshops generated an interest in reaching more people through the development of an online M&E for the malaria course, which was launched in 2012. An open-sourced curriculum is also available online in English [20]. An online course will be launched in French in 2016 [21].

Results from the overall post-workshop assessment revealed that participants retained practical M&E knowledge and skills. One participant remarked, “Immediately after the workshop, I was part of a team that was assigned to review our project’s M&E plan…and most of my contributions to the process were based on what I learned from the workshop. To be specific, when it came to developing an indicator reference sheet/matrix, we actually used the template developed by my workshop’s group members as an example.”

There was positive feedback about the workshops from participants, supervisors, and stakeholders related to the relevance of the course content, application of knowledge and skills gained, improvement of overall M&E capacity, and change in perception/support of M&E activities.

“We used to have some discrepancies in data and reporting, but using an online data management software along with training of the staff in M&E, after having the training, the quality of data has been improved remarkably in data collection and reporting to mention few.”

There was a willingness to continue supporting the regional M&E capacity of malaria workshops “…especially for some of us who live in countries that do not have institutions that provide such courses, for those who cannot afford to pay school fees, and for the convenience of being able to do it at my own time. This combination makes this platform an excellent initiative.”

Further findings identified a need for new modules to be created, particularly malaria surveillance and evaluation methods, to add more value to the course and identify the changing needs in malaria programmes. There was also an urgent need to establish a more robust post-training follow-up programme and analyse the participants’ costs associated with the course.

## Discussion

This paper documents efforts to build capacity within NMCPs for malaria M&E through workshops for malaria-endemic countries in SSA from 2010 to 2014. The workshops contributed to training a significant number of health professionals involved in malaria control and elimination efforts who are now contributing to improve malaria M&E in their respective countries. The design, content, and implementation process of these workshops facilitated the buy-in and interest from countries and key partners involved in the malaria control efforts at the national, regional, and global levels.

Establishing strong country partnerships is essential to the success of capacity building interventions. UGSPH and CRSN assembled a cohesive group of experienced facilitators to teach the workshops and created links between institutions inside and outside host countries. Facilitators at the Anglophone course were from UGSPH, the Ghana National Malaria Control Programme, the Ghana Health Service, the World Health Organization’s country office, and the Noguchi Medical Centre. Similarly, the Francophone course included facilitators from Centre de Recherche en Santé de Nouna (CRSN), Centre de Recherche Internationale pour la Santé (CRIS)/University of Ouagadougou, Centre Muraz, the Burkina Faso National Malaria Control Programme, and the Burkina Faso Ministry of Health. Each facilitator brought extensive years of M&E experience to enrich the curriculum.

The importance of regular communication with previous workshop cohorts to guide future participation is vital to the success of forthcoming capacity-building workshops. This communication has generated new country-specific M&E trainings to improve M&E malaria capacity at the national level. Course alumni have been actively involved as facilitators and key promoters of these courses. Alumni have also contributed to impact evaluations and other M&E malaria control and pre-elimination activities at the country level upon completion of the workshops.

Though the success of this capacity-building intervention can be measured individually through pre- and post- workshop tests, evaluations, and follow-up surveys, it is challenging to measure the contribution to the improvement of country-level malaria M&E system. Workshop alumni changed positions; completed additional training courses; and worked in multiple programmes like HIV/AIDS, tuberculosis, and others. This further complicates measurements of the contributions of this one malaria capacity-building intervention to the improvement of countries’ malaria information systems and health information systems overall. However from the alumni feedback, the skills gained from the training are being applied to improve data quality and reporting.

While this workshop model trained 181 M&E professionals from 28 countries on the fundamentals needed to improve their respective country-level malaria information systems, capacity gaps were still identified. Francophone participants had a more difficult time securing funding than Anglophone participants. Workshop costs were too high for the majority of participants to self-fund. In 2014, the courses cost $3000 plus travel for the Anglophone course and $3870 for the Francophone course. These costs included tuition, course materials, housing, and breakfast and lunch for the duration of the workshop, and they were designed to provide a small margin of profit to support partner institutions. Although, a few fellowships are available every year, most participants had to rely on a sponsor. The length of the workshop is also in question, as there is not consensus on what is the optimal training length to learn and retain the training materials. Workshop management costs were linked to only one funding source, which has fiscal and project year limitations. Regional university venues provided an established professional learning environment, but infrastructure issues were abundant.

## Conclusion

The results of the post-workshop assessment provide a good way forward for the future of these workshops [22]. Recommendations from participants, supervisors, and stakeholders will guide the next phase of workshops in the curriculum development and restructuring of the workshops. Details are currently under discussion, including revamping the current M&E fundamentals curriculum to include optional tracks on targeted M&E topics. Such topics may include data presentation, interpretation, and use; data analysis; surveillance; and impact evaluation. Additional ideas include contributing to a malaria module for the anticipated one-year Master’s programme in M&E at the University of Ghana. However, country malaria M&E capacity needs assessments may be necessary to inform this process, especially as countries shift from malaria control to pre-elimination to elimination. Finally, the sustainability of these workshops and any capacity-building activities in malaria M&E will depend on the availability of funding and countries being able to tap into existing funding mechanisms.
